# A Large-Scale Sensor Layout Optimization Algorithm for Improving the Accuracy of Inverse Finite Element Method

**DOI:** 10.3390/s23198176

**Published:** 2023-09-29

**Authors:** Zhenyi Zhao, Kangyu Chen, Yimin Liu, Hong Bao

**Affiliations:** 1 Key Laboratory of Electronic Equipment Structure Design, Ministry of Education, Xidian University, Xi’an 710071, China; 22041212766@stu.xidian.edu.cn (Z.Z.); ky.chen@stu.xidian.edu.cn (K.C.); 22041212778@stu.xidian.edu.cn (Y.L.); 2 Intelligent Robot Laboratory, Hangzhou Research Institute of Xidian University, Hangzhou 311231, China

**Keywords:** inverse finite element method, cooperative coevolution, particle swarm optimization, grouping method, structural health monitoring

## Abstract

The inverse finite element method (iFEM) based on fiber grating sensors has been demonstrated as a shape sensing method for health monitoring of large and complex engineering structures. However, the existing optimization algorithms cause the local optima and low computational efficiency for high-dimensional strain sensor layout optimization problems of complex antenna truss models. This paper proposes the improved adaptive large-scale cooperative coevolution (IALSCC) algorithm to obtain the strain sensors deployment on iFEM, and the method includes the initialization strategy, adaptive region partitioning strategy, and ***gbest*** selection and particle updating strategies, enhancing the reconstruction accuracy of iFEM for antenna truss structure and algorithm efficiency. The strain sensors optimization deployment on the antenna truss model for different postures is achieved, and the numerical results show that the optimization algorithm IALSCC proposed in this paper can well handle the high-dimensional sensor layout optimization problem.

## 1. Introduction

In recent decades, the application of structural health monitoring (SHM) has gained considerable attention in various fields such as large-scale bridges, high-rise buildings, and aerospace. The practical implementation techniques have also been investigated by engineering researchers [1,2,3,4]. SHM provides a strong technical guarantee for structural safety through real-time monitoring and analysis of various structural data, timely detection of structural defects and hidden dangers, and prediction of structural life and fatigue state. In Gherlone’s research on deformation reconstruction methods [5], shape sensing was utilized to reconstruct the deformation field of a structure by measuring real-time strain values using strain sensors attached to different positions of the structure.

The key aspect of shape sensing is to establish an accurate mathematical relationship between the measured surface strain values and the reconstructed displacement field. Various modeling approaches have been proposed by researchers for this purpose. Some authors have employed methods such as fuzzy networks and neural networks for modeling [6,7]. These approaches require a substantial amount of training samples and the quality of these samples affects the accuracy of the reconstruction. In the literature [8,9], the surface-measured strain is fitted to obtain the structural strain field using global or segmented continuous basis function methods. Subsequently, the displacement field of the structure is obtained through the strain–displacement relationship. This approach is easily implementable, but the accuracy of the reconstruction relies on the selection of basic functions and weight coefficients. Jineesh et al. selected modal shapes as basic functions and reconstructed the displacement field using modal transformation techniques [10,11]. This method exhibits better reconstruction accuracy due to its inherent modal characteristics. However, the drawback lies in the requirement of detailed material properties for constructing modal shapes, making it less applicable to complex structures. 

Tessler and Spangler proposed the inverse finite element method (iFEM) based on the first-order shear deformation theory [12,13]. This method offers the advantage of accomplishing deformation reconstruction without requiring prior knowledge of load, material parameters, and structural properties. The framework of the iFEM was extended by Gherlone for the beam element, who validated the feasibility of their approach by conducting shape sensing analysis on a cantilever beam structure subjected to static and dynamic loads [14]. In [15], shape sensing analysis was conducted on a typical composite reinforced structure using the iFEM, demonstrating the superiority of the iFEM. Data collection from individual surfaces of the structure was employed by Niu et al. to reconstruct the displacement field [16], thus reducing the number of sensors used. Chen et al. introduced a unified displacement field for beam-like structures based on the homogenization theory, utilizing certain generalized quantities instead of traditional displacement functions to avoid reconstruction errors caused by structural identification inaccuracies [17]. Since the measured strain values from strain sensors serve as crucial data for shape sensing techniques, the layout positions of strain sensors have a significant impact on the reconstruction accuracy. Feng et al. proposed a method based on numerical optimization to obtain the best sensor position [18]. Zhao et al. optimized the layout of sensors using a single-objective particle swarm optimization (SOPSO) algorithm, targeting well-separated eigenvalues [19]. However, using a SOPSO algorithm makes it challenging to strike a balance between robustness and accuracy. In [20], the optimization was performed using a multi-objective particle swarm algorithm (MOPSO) with robustness and accuracy as optimization objectives, but the absence of an appropriate strategy to ensure diversity resulted in a tendency to get trapped in the local optima. By introducing strategies such as guided particle selection and maintenance of an external candidate solution set, Li et al. improved the optimization performance [21]. The above algorithms improve the optimization by introducing different methods, but the effectiveness of these methods significantly diminishes when the dimensionality of the solution space becomes too large. 

Optimizing sensor layout with reconstruction accuracy and robustness as optimization objectives constitutes a multi-objective optimization problem (MOP). Currently, the adoption of cooperative coevolution (CC) is considered as a successful approach for addressing large-scale multi-objective optimization problems (LSMOPs). The CC method was proposed by Potter and De Jong in [22], where the idea is to decompose the decision variables using various variable grouping methods and optimize them independently. The CC framework is primarily composed of three components: grouping strategy, optimizer, and collaboration method. The grouping strategy is a method for decomposing a large number of decision variables, and existing grouping strategies include fixed grouping; random grouping [23]; linear grouping [24]; ordered method [25]; and dynamic grouping [26]. These groupings are usually simpler, but since they do not take into account the attributes of the decision variables, their enhancement effect on optimization is small. The optimizer is the algorithm used to optimize each subgroup, such as the aforementioned MOPSO algorithm [20,21]; they perform poorly when faced with high-dimensional optimization problems. The collaboration method defines the inter-group collaboration strategy, which specifies the content and manner of information sharing between subgroups [27]. The objective of this paper is to propose an optimization algorithm that can be applied to large-scale sensor layout optimization problems.

This paper improves and optimizes the above two parts respectively. In terms of grouping strategy, citing the concept of the convergence relevance degree (CRD) proposed by Ma et al. in [28], on the basis of calculating the CRD value of each decision variable, a grouping method according to the size of the CRD value is proposed; this method can make full use of the attribute characteristics of decision variables and attribute decision variables with similar characteristics to the same subgroup. In terms of the optimizer, in order to balance the diversity and convergence of the population and prevent the optimization result from falling into a local optimum, a method of adaptive region division based on the number of external archive particles is designed, and particle updates in each region are guided by the globally optimal particles in other regions. The advantage of this method is that it can fully explore the target space during the optimization process and improve the search efficiency by limiting the number of non-dominated solutions in the external archive. 

The organization of this paper is as follows. In Section 2, the iFEM for the basic beam element is introduced, and a sensor layout optimization model based on reconstruction accuracy and robustness is established. In Section 3, the improved adaptive large-scale cooperative coevolution (IALSCC) algorithm is described in detail. In Section 4, IALSCC is applied to the sensor layout problem for deformation reconstruction of complex antenna truss structure, and simulation data verifies that IALSCC is a promising tool for sensor layout optimization. 

## 2. Optimization Model Based on iFEM for Beam Structures

### 2.1. Inverse Finite Element Method 

**Figure 1:**
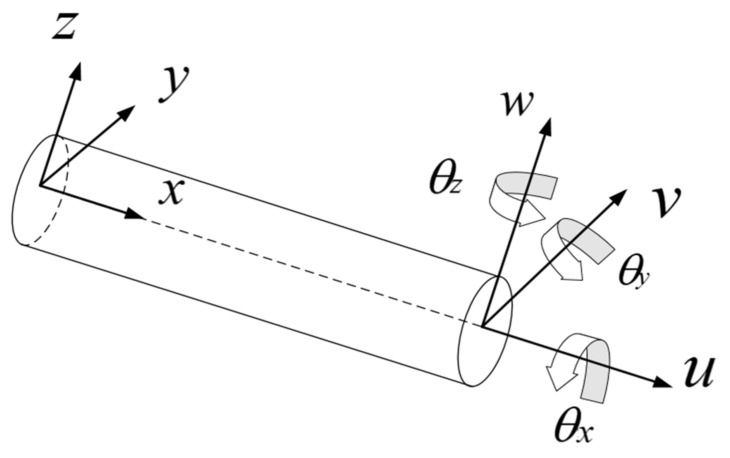
Schematic diagram of Timoshenko beam.

According to the Timoshenko beam theory, as shown in Figure 1, the deformation of any point on the surface of the beam can be represented by displacements along the respective axes and rotations about the respective axes. These six kinematic variables can be grouped in vector form:

(1) $$u={\left[u,v,w,\theta_{x},\theta_{y},\theta_{z}\right]}^{T}.$$

Based on the kinematic assumption of three-dimensional deformation, the displacement vector at any point on the beam cross-section can be represented by the displacement of the neutral axis:

(2) $$\;\;\;\;\;\;\;\;\;\left\{\begin{array}{c} u_{x}\left(x,\;y,z\right)=u\left(x\right)+z\theta_{y}\left(x\right)-y\theta_{z}\left(x\right)\\ u_{y}\left(x,y,z\right)=v\left(x\right)-z\theta_{x}\left(x\right)\\ u_{z}\left(x,y,z\right)=w\left(x\right)+y\theta_{x}\left(x\right) \end{array}\right..$$

Based on the assumption of small strains, the strain vector at any cross-section is defined as

(3) $$e\left(u\right)={\left[e_{1},e_{2},e_{3},e_{4},e_{5},e_{6}\right]}^{T},$$

where $e_{1}$ is the cross-sectional strain caused by the elongation deformation of the element, $e_{2}$ and $e_{3}$ are the cross-sectional strains caused by the bending deformation of the element, $e_{4}$ and $e_{5}$ are related to the shear deformation, and $e_{6}$ is related to the torsional deformation of the element. The cross-sectional strains can be obtained from Equation (2):

(4) $$\left\{\begin{array}{c} e_{1}\left(x\right)=u_{x}\left(x\right),\;e_{2}\left(x\right)=\theta_{y,x}\left(x\right)\\ e_{3}\left(x\right)=-\theta_{z,x}\left(x\right),\;e_{4}\left(x\right)=w_{x}\left(x\right)+\theta_{y}\left(x\right)\\ e_{5}\left(x\right)=v_{x}\left(x\right)-\theta_{z}\left(x\right),\;e_{6}\left(x\right)=\theta_{x,x}\left(x\right) \end{array}\right..$$

The strain along the axis at any point is obtained by taking the derivative of Equation (2) with respect to x:

(5) $$\left\{\begin{array}{c} \varepsilon_{x}\left(x,y,z\right)=e_{1}\left(x\right)+ze_{2}\left(x\right)+ye_{3}\left(x\right)\\ \gamma_{xz}\left(x,y\right)=e_{4}\left(x\right)+ye_{6}\left(x\right)\;\\ \gamma_{xy}\left(x,y\right)=e_{5}\left(x\right)-ze_{6}\left(x\right) \end{array}\right..$$

The basic framework of the iFEM is to minimize the error between experimental and theoretical strains using the least squares method. The fitting function $\varphi \left(u\right)$ is defined by the theoretical sectional strain $e\left(u\right)$ and the actual sectional strain $e^{\varepsilon }$:

(6) $$\varphi \left(u\right)=\|e\left(u\right)-e^{\varepsilon }\|{}^{2}.$$

By taking the derivative of Equation (6) with respect to u and setting the derivative equal to 0, the relationship between the neutral axis displacement variable of the beam and the experimental strain measurements is established:

(7) $$k^{e}u_{e}=f^{\varepsilon }.$$

$k^{e}$ and $f^{\varepsilon }$ can be expressed as

(8) $$\left\{\begin{array}{c} \;k^{e}=\sum_{k=1}^{6}w_{k}k_{k}^{e},k_{k}^{e}=\sum_{i=1}^{n}\frac{L}{n}\left[B_{k}^{T}\left(x_{i}\right)B_{k}\left(x_{i}\right)\right]\\ f^{\varepsilon }=\sum_{k=1}^{6}w_{k}f_{k}^{e},f_{k}^{e}=\frac{L}{n}\sum_{i=1}^{n}\left[B_{k}^{T}\left(x_{i}\right)e_{k}^{\varepsilon }\left(x_{i}\right)\right] \end{array}\right.,$$

where n represents the number of sections, L is the length of the element, $x_{i}$ is the calculation position of the strain, $e_{k}^{\varepsilon }\left(x_{i}\right)$ is the calculated strain at position $x_{i}$ obtained from the measured strain data, $w_{k}\left(k=1,2,\cdots ,6\right)$ are the weighting coefficients that consider the mutual influence of axial stretching, bending, torsion, and transverse shear, $B_{k}\left(x_{i}\right)$ is the coefficient matrix obtained by taking the derivative of the shape function matrix, and once $x_{i}$ is determined, the coefficient matrix is also determined. It can be observed that $k^{e}$ is a function of $x_{i}$, and $f^{\varepsilon }$ is determined by $x_{i}$ and the measured strain value $\varepsilon_{2}^{*}$.

Equation (9) is obtained by assembling multiple inverse finite element units:

(9) KU=F,

where K is the overall class stiffness matrix, determined by the shape functions and the positions of strain sensors, and F is the global class load vector, which depends only on the measured strain. This equation provides the solution for the degrees of freedom of the structural deformation nodes, $U=K^{-1}F$. Then, since the displacement at any cross-section location can be expressed using shape functions and nodal degrees of freedom, the deformation at any cross-section can be obtained using interpolation methods. Finally, based on Equation (2), the deformations at arbitrary points can be calculated.

### 2.2. Metrics to Evaluate Refactoring Effects

After reconstructing the displacement field of the structure, it is necessary to evaluate the performance of the reconstructed field. In this study, the root mean square error (*RMSE*) and robustness index (*RBI*) are employed to assess the accuracy of the reconstruction and the stability of the reconstruction model, respectively.

Reconstruction accuracy is a key indicator for evaluating the effectiveness of the iFEM, is measured by the *RMSE*, and defined as follows:

(10) $$RMSE\left(X\right)=\sqrt{\frac{1}{N}\sum_{i=1}^{N}{\left(disp_{i}^{measurement}\left(X\right)-disp_{i}^{iFEM}\left(X\right)\right)}^{2}},$$

where X represents the sensor layout scheme, N is the number of reference points, $disp^{measurement}$ is the actual measured value obtained through simulation software or measurement devices, $disp^{iFEM}$ is the predicted value derived using the iFEM, and $disp\left(X\right)$ is the deformation displacement along the centroid axis. The key step of iFEM is to calculate the cross-section strain based on the measured surface strain data, and the placement of the strain sensor directly affects the value of the surface strain and the calculation of the class stiffness matrix, which in turn affects the reconstruction accuracy of the displacement field.

Robustness is also an important indicator for evaluating the effectiveness of deformation reconstruction, which reflects the ability of the model to maintain performance stability in the face of uncertainty and perturbations. In order to clearly represent this ability and optimize it as an objective, a method for quantifying reconstruction stability introduced in [29] is employed.

(11) $$cos\left(\theta_{i}\left(X\right)\right)=\left|\frac{A_{i}\left(X\right)P\left(X\right)A_{i}^{T}\left(X\right)}{\left\|A_{i}\left(X\right)\|\times \|P\left(X\right)A_{i}^{T}\left(X\right)\right\|}\right|,$$

(12) $$P_{i}\left(X\right)={\bar{A}}_{i}^{T}\left(X\right){\left({\bar{A}}_{i}\left(X\right){\bar{A}}_{i}{}^{T}\left(X\right)\right)}^{-1}{\bar{A}}_{i}\left(X\right),$$

where the matrix $A\left(X\right)$ is a class stiffness matrix that is only dependent on the sensor layout. $A_{i}\left(X\right)$ represents the i-th row vector of matrix $A\left(X\right)$, while ${\bar{A}}_{i}\left(X\right)$ is a matrix composed of the remaining row vectors excluding $A_{i}\left(X\right)$. The larger the angle $\theta_{i}\left(X\right)$ becomes, the lower the correlation between the vector matrices, indicating a stronger ability to withstand external perturbations. By calculating the angle $\theta_{i}\left(X\right)$ for each row vector, the *RBI* is defined as the minimum value among them:

(13) $$RBI\left(X\right)=\min\left(\theta_{i}\left(X\right)\right)\;i=1,2,\cdots ,m,$$

where m represents the dimension of the node degrees of freedom. The larger the $RBI\left(X\right)$ value, the stronger the anti-interference ability of this sensor layout scheme.

After defining the two objective functions, the sensor layout scheme that balances reconstruction accuracy and robustness can be sought through an optimization algorithm. The optimization model is defined as follows:

(14) $$X=argmin\;\left\{RMSE\left(X\right),-RBI\left(X\right)\right\},$$

where X represents the layout positions of the sensors with dimensions equivalent to the number of sensors. In the third section, the proposed IALSCC algorithm will be thoroughly described.

## 3. Improved Adaptive Large-Scale Cooperative Coevolutionary Algorithm

The algorithm proposed in this paper focuses on optimizing and improving two components: the optimizer and the grouping method. The optimizer is a MOPSO algorithm, while the grouping method addresses the issue of grouping a large number of decision variables. In Section 3.1, the MOPSO algorithm will be introduced. In Section 3.2, the improved multi-objective particle swarm optimization algorithm proposed in this paper will be discussed. Section 3.3 will present the grouping method based on CRD calculation and difference degree (DD). Finally, Section 3.4 outlines the algorithm framework of IALSCC.

### 3.1. Introduction of MOPSO

PSO is an intelligent algorithm inspired by the foraging behavior of birds, originally proposed by Kennedy and Eberhart in [30]. The principle is that each particle is guided to update itself based on its own historical information and the information of the swarm. The expression of the algorithm is given by the following equations:

(15) $$v_{id}^{t+1}=\omega v_{id}^{t}+c_{1}r_{1}\left(pbest_{id}^{t}-x_{id}^{t}\right)+c_{2}r_{2}\left(gbest_{d}^{t}-x_{id}^{t}\right),$$

(16) $$x_{id}^{t+1}=x_{id}^{t}+v_{id}^{t+1},$$

where $v_{id}^{t}$ is the d-th component of the velocity vector of particle i at the t-th iteration; $x_{id}^{t}$ is the d-th component of the position vector of particle i at the t-th iteration; $pbest_{id}^{t}$ is the d-th component of the optimal position vector of particle i at the t-th iteration; $gbest_{d}^{t}$ is the d-th component of the optimal position vector of the population at the t-th iteration; $c_{1}$ and $c_{2}$ are self-awareness coefficient and group learning coefficient, respectively; $r_{1}$ and $r_{2}$ are two random numbers, the value range is $\left[0,1\right]$; and ω is a inertia weight, which adjusts the search range of the solution space.

The MOPSO algorithm is an optimization algorithm proposed based on the PSO algorithm to address MOPs [31]. A MOP can be described as a minimization problem, defined as follows:

(17) $$Minimize\;F\left(X\right)=\left\{F_{1}\left(X\right),F_{2}\left(X\right),\cdots ,F_{n}\left(X\right)\right\}\;\mathrm{subject}\;\mathrm{to}\;X\in \Omega$$

where n is the dimension of the objective space and Ω is the decision variable space. Obviously, the existence of multiple objective functions makes it difficult to compare the quality of solutions. The Pareto dominance relation is a commonly used method for comparing the quality of solutions, which is defined as if $X,Y\in \Omega ,\;\forall k\in \left\{1,2,\cdots ,m\right\},\;F_{k}\left(X\right)\leq F_{k}\left(Y\right)$ and $\exists k\in \left\{1,2,\cdots ,m\right\},\;F_{k}\left(X\right)<F_{k}\left(Y\right)$; m is the number of decision variables and Y is said to be dominated by X, otherwise there is a non-dominated relationship between X and Y.

The balance between diversity and convergence is a crucial aspect in ensuring the effectiveness of the MOPSO. If emphasis is placed on convergence while neglecting exploration of the objective space, the results may easily become trapped in local optima. Conversely, if focus is solely on exploration without sufficient convergence, the exploration of the objective space may prove ineffective. The proposed improved algorithm in this paper effectively balances these two aspects and demonstrates excellent performance in handling MOPs.

### 3.2. Strategies Used by the Proposed Improved MOPSO

#### 3.2.1. Initialization Strategy

In traditional MOPSO algorithms, the velocity and position of particles are typically randomly initialized. In this algorithm, the initial region partitioning in the objective space is based on the distribution of particles. Uneven distribution of particles can result in a low number of particles entering the optimization process, leading to decreased exploration efficiency. Therefore, it is necessary to improve the initialization strategy to achieve a more uniform distribution of initial particles in the objective space.

Using boundary particles as global best particles to guide the population for early-stage updates is an effective method. Specifically, assuming there are n optimization objectives, during the initialization stage, n rounds of single-objective optimization are performed. It is important to note that after completing each round of single-objective optimization, the results of each iteration in that round are saved. Then, the next single-objective optimization should begin from the initial state rather than from the previous optimization results. The final set of particles is the union of the saved results. Additionally, the number of iterations T_ini and the initial population size N_ini need to be appropriately chosen. If the parameters are not suitable and result in a large population size N, it can affect the efficiency of the algorithm. Conversely, if N is too small, the uniformization effect may not be satisfactory. Generally, N_ini<N/5 and T_ini<T/10 are considered appropriate, where T is the maximum number of iterations.

By employing this initialization strategy, the distribution of particles in the objective space can be made more uniform, thus enhancing the population diversity and the performance of the optimizer in MOPSO.

#### 3.2.2. Adaptive Region Partitioning Strategy

In this paper, a method is proposed for adaptive region partitioning based on the distribution of particles in an external archive set. Taking bi-objective optimization as an example, after uniform initialization of particles, non-dominated solutions are selected based on the Pareto dominance relation and stored in the external archive set.

The external archive set is divided into $D_{i}$ regions based on the given number of regions, and it is important to note that normalization of the objective space is performed during the region partitioning to prevent some particles from being neglected in the calculation.

**Figure 2:**
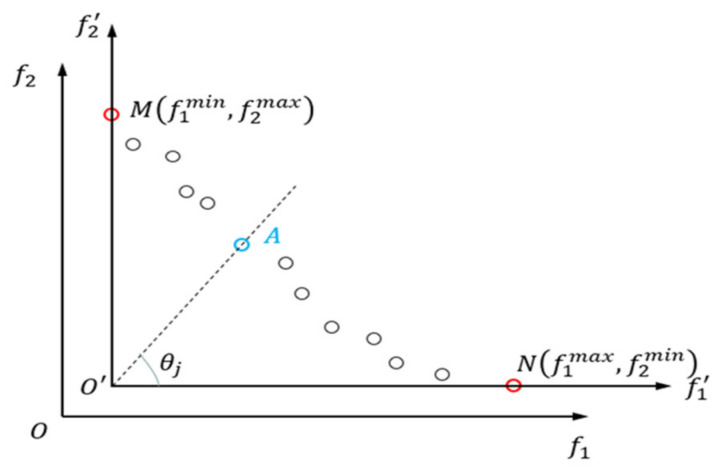
Normalized coordinate establishment.

As shown in Figure 2, when optimizing the objective $f_{1}$, point M has the minimum fitness value $f_{1}^{min}$. When optimizing the objective $f_{2}$, point N has the minimum fitness value $f_{2}^{min}$. After normalization, the coordinate origin shifts from the original point O to $O^{\prime }\left(f_{1}^{min},f_{2}^{min}\right)$. Then, in the normalized coordinate system, the angle $\theta_{j}$ between the line connecting the non-dominated particle and the origin and the horizontal axis is calculated using Equation (18):

(18) $$\theta_{j}=arctan\left(\left(f_{2}\left(x_{j}\right)-f_{2}^{min}\right)/\left(f_{1}\left(x_{j}\right)-f_{1}^{min}\right)\right),$$

where $f_{2}\left(x_{j}\right)$ represents the fitness of the j-th particle in optimization objective $f_{2}$ and $f_{1}\left(x_{j}\right)$ represents the fitness of the j-th particle in optimization objective $f_{1}$. Finally, based on Equation (19), it is determined whether each region contains particles. For regions that contain particles, one particle is selected from each region, and their union forms the population for formal optimization. These particles represent high-quality information from the initial population, and utilizing them can improve the efficiency of the algorithm. Equation (19) is as follows:

(19) $$0.5\times \pi \times \left(i-1\right)/D_{i}\leq \theta_{j}<0.5\times \pi \times i/D_{i},$$

where i represents the i-th region and $D_{i}$ represents the total number of divided regions.

After the aforementioned steps, we can calculate that among the $D_{i}$ regions in the external archive set there are space_n regions that already contain non-dominated solutions. Then, based on the range of space_n, the objective space is divided into $D_{j}\in \left[1,4\left(2^{n}\right)\right]$ regions. The specific partitioning method is as follows.

If $0<space\_n\leq 1/4\times D_{i}$, then $D_{j}=4$. In this case, the value of space_n is small, indicating that non-dominated particles are not present in most regions. There are two possibilities: one is that non-dominated solutions do not exist in that region, indicating that the Pareto Front (PF) is discontinuous; the other is that the region has not been explored, suggesting insufficient population diversity, which requires enhancing exploration in that region to improve diversity. If $1/4\times D_{i}<space\_n\leq 2/4\times D_{i}$, then $D_{j}=3$; if $2/4\times D_{i}<space\_n\leq 3/4\times D_{i}$, then $D_{j}=2$; if $3/4\times D_{i}<space\_n\leq 1\times D_{i}$ and $D_{j}=1$. In the latter case, where most regions already meet the condition of having non-dominated solutions, there is no need for further processing of the objective space.

After the initial region partitioning, it is necessary to find gbest to guide particle updates. With each iteration process completed, the value of space_n is recalculated and the objective space is adaptively partitioned based on the value of space_n. The selection strategy for gbest will be described in detail in the next section.

#### 3.2.3. Selection of gbest and Particle Update Strategies

The selection of gbest has a significant impact on the optimization results. In previous algorithms, typically only one gbest is selected in the objective space to guide the optimization process, aiming to ensure convergence. However, in high-dimensional optimization problems, due to the large number of decision variables, the solution space becomes rapidly complicated and it is more likely that the optimization result will fall into the local optimum. Therefore, on the basis of considering convergence, this method places more emphasis on improving population diversity.

**Figure 3:**
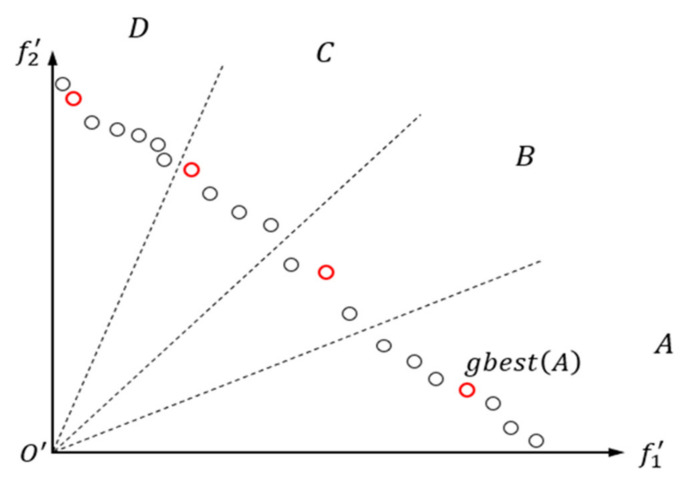
Gbest selection and particle update strategies ($D_{j}=4$).

In Section 3.2.2, the objective space has been divided into $D_{j}$ regions, taking $D_{j}=4$ as an example. A non-dominated solution is randomly selected from each of the four regions as the gbest for that region. Then, the gbest of each region is used to guide the particle updates in other regions. The purpose of this operation is to allow particles to thoroughly explore the objective space and obtain potentially more promising non-dominated solutions. As shown in Figure 3, $gbest\left(A\right)$ is randomly selected in region A, and it is used to guide the particle updates in regions other than A (e.g., region D). It should be noted that if there are no particles in a particular region, resulting in the absence of gbest for that region, the gbest from the adjacent region is used as a substitute. The advantage of this approach is its ability to thoroughly explore the objective space, significantly enhancing the diversity of the population.

After each iteration, the non-dominated particles are saved to the external archive. Then, based on the Pareto dominance relationship, all non-dominated particles in the external archive are filtered out. The external archive contains all the non-dominated particles found so far in the search. It is important to note that as the iterations progress, the number of non-dominated solutions in the external archive grows rapidly. Therefore, it is necessary to set a threshold to prevent a decrease in algorithm efficiency. The threshold needs to be properly set, as a too-low threshold may result in a high deletion frequency of particles, potentially leading to the removal of some high-quality particles. On the other hand, a too-high threshold can significantly decrease the efficiency of the algorithm. Once the number of non-dominated solutions exceeds the threshold, the distribution of particles in the $D_{i}$ regions within the external archive is examined. In regions where multiple particles exist, only one particle is retained and the rest are deleted. This process is repeated until the maximum number of iterations is reached or non-dominated particles exist in all $D_{i}$ regions.

### 3.3. Grouping Method Based on CRD Calculation and DD

#### 3.3.1. Calculation Method of CRD

**Figure 4:**
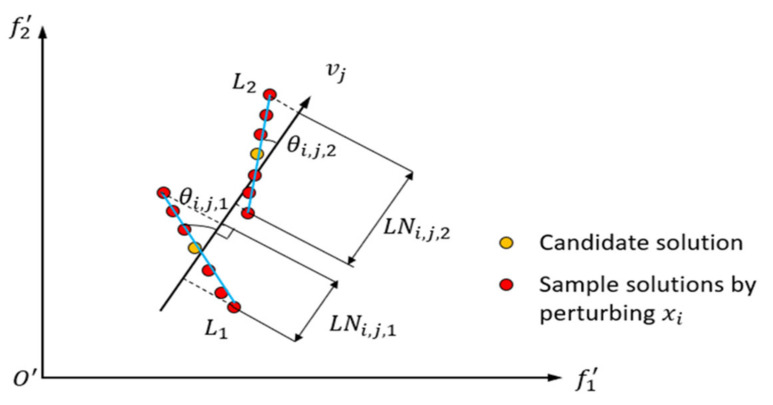
Schematic diagram of the parameters of the CRD calculation method (T=2).

In [26], a method for quantitatively calculating the CRD of decision variables is proposed. It involves the following steps:

(1) Normalize the objective space and generate the reference vector $v_{j}$; (2) For each reference vector, its neighboring solutions are determined by $Neighbor\left(T\right)$, which represents the set of solutions closest to that reference vector among all candidate solutions. Therefore, it is necessary to calculate the angle between each candidate solution and the reference vector and select the T closest candidate solutions. The angle is calculated using the following formula:

(20) $$angle\left(s,v_{j}\right)=arccos\left(\frac{F^{T}\left(s\right)v_{j}}{\left\|F\left(s\right)\|\|v_{j}\right\|}\right)$$

where s represents a candidate solution and $F\left(s\right)$ represents the connecting vector between the candidate solution and the normalized origin; (3) For each of the T candidate solutions, k perturbations are added to each dimension and the generated sampled solutions are fitted into a line. The projection $LN_{i,j,T}$ of these solutions on the reference vector is calculated. The computation of CRD is related to the length of the projection and the angle, and the specific calculation formula is as follows:

(21) $$CRD_{i,j}=\left(1+\frac{\theta_{i,j}-\theta_{min}}{\theta_{max}-\theta_{min}}\right)e^{-LN_{i,j}}$$

where $\theta_{i,j}$ represents the angle between the fitted line and the reference vector and $LN_{i,j}$ denotes the length of the projection of the fitted line on the reference vector. $\theta_{min}$ and $\theta_{max}$, respectively, represent the minimum and maximum values among all the angles between candidate solutions and the reference vector, as shown in Figure 4. In the end, for each reference vector, T CRDs are calculated for each dimension and their average is computed as a measure of the convergence relevance for that dimension.

#### 3.3.2. Grouping Strategy Based on DD

After the calculation of CRD, the decision variables should be divided into multiple subgroups. The number of subgroups should not be too small relative to the number of decision variables to avoid having some subgroups with a large number of variables, which would result in ineffective grouping. Therefore, a strategy is proposed to group the variables based on the DD. This strategy automatically determines the number of subgroups and their sizes.

The CRD values of the decision variables are sorted in descending order, and the maximum value, $crd_{max}$, and the minimum value, $crd_{min}$, in this sequence are identified. Based on the predefined minimum number of subgroups, $sub_{min}$, the difference threshold is defined as follows:

(22) $$DT=\left(crd_{max}-crd_{min}\right)/sub_{min}.$$

Then, the DD of adjacent values in the CRD sequence is calculated as follows:

(23) $$DD_{i}=crd_{i}-crd_{i-1},$$

where i represents the index of the CRD sequence. If $DD_{i}\leq DT$, the decision variable is placed in the current subgroup. If $DD_{i}>DT$, the decision variable is placed in a new subgroup. This process is repeated until all decision variables are grouped. According to the calculation principle of CRD, the smaller-numbered subgroups contribute more to diversity. The overall pseudocode for the adaptive grouping based on the CRD method is shown in Algorithm 1.
**Algorithm 1:** Framework based on CRD and DD grouping**Input**: **X**: particles after initialization; **fx 1** and **fx 2**: objective function values *RMSE* and *RBI*; **N**: number of particles; **D**: number of decision variables; **xlimit**: particle position boundary; **sub_min_**: minimum number of groups. 1. Set the reference point and obtain the reference vector $v_{j}$. 2. Calculate the angle between each particle and each reference vector. 3. Select the two particles with the smallest angle as $Neighbor\left(T\right).$ 4. Add perturbation in each dimension, fit the generated sampling solution to a straight line, and calculate the angle and projection ($\theta_{i,j}$ and $LN_{i,j}$) between the straight line and the reference vector. 5. Calculate CRDs according to Formula (21). 6. Grouping decision variables based on DD. **Output: Group_num:** number of groups; **Subgroup:** decision variables within subgroups.

### 3.4. Algorithm Framework

**Figure 5:**
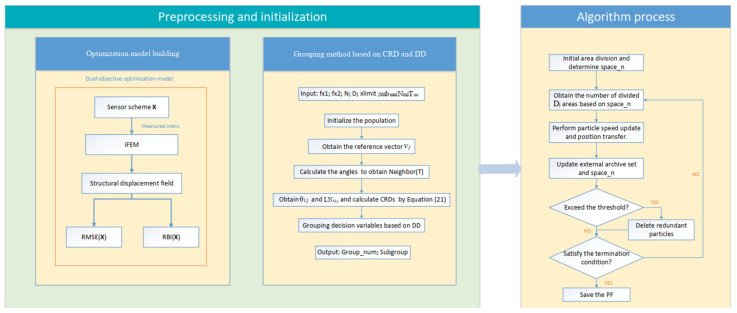
Algorithm Flowchart.

The framework of IALSCC is described as follows (see Algorithm 2), and Figure 5 illustrates the flowchart of the proposed IALSCC algorithm for sensor layout optimization, which is divided into three steps: model building, variable grouping, and optimization.
**Algorithm 2**: Framework of IALSCC**Input:** $N_{ini}$: number of initial particles; $T_{ini}$: number of initial particle iterations; **iter**: the maximum number of iterations; **D**: number of decision variables; **xlimit**: particle position boundary; **vlimit**: particle velocity boundary; **loop**: co-evolution times;
ω: inertia weight;
$c_{1}$ and $c_{2}$: self-awareness coefficient and group cognitive coefficient; $D_{i}$; number of divisions; ${sub}_{min}$: minimum number of groups. **Termination condition:** the maximum number of iterations is reached or region particle completeness is satisfied. **Step 1: Initialization** 1. Using $N_{ini}$ and $T_{ini}$, perform two single-objective optimizations on the two objective functions. 2. Grouping decision variables using CRD and DD. 3. Using the initial region division strategy, filter out the non-dominated solutions and save them in the external file and then obtain the number of regions (space_n) that satisfy the particle existence condition. **Step 2: Iteration** **For**
i←1 to loop **do** **For**
j←1 to Group_num **do**    1.The number of divided areas $D_{j}$ is determined by the range of space_n.   2.Randomly select a particle in each region as the gbest of the region.   3.Perform particle speed update and position transfer.   4.The non-dominated solutions are screened out from the updated particles and added to the external archives, and the non-dominated solutions need to be screened again in the external archive.   5.Calculate the number of areas in the external file that satisfy the existence of particles and update space_n.   6.Determine whether the particles in the external archive exceed the threshold and delete redundant particles if the threshold is exceeded.   7.Judging whether the termination condition is satisfied. If so, output the external archive; otherwise, enter the next iteration. **END** **END** **Output:** external archive.

## 4. Algorithm Evaluation

### 4.1. Algorithm Effect Verification

To validate the optimization effectiveness of the algorithm, the grouping method proposed in this paper based on CRD and DD will be compared with other grouping methods, demonstrating the superiority of this grouping approach. The improved optimizer will be compared with AADMOPSO presented in [32], to demonstrate the effectiveness of the optimizer’s improvements. The ZDT1, ZDT2, ZDT3, and ZDT6 test problems from the ZDT benchmark suite are selected as validation models. ZDT1 exhibits a continuous and uniformly distributed PF, ZDT2 features a non-convex PF, ZDT3 consists of five non-convex PFs, and ZDT6 has a non-uniformly distributed and non-convex PF [33]. The algorithm’s convergence and diversity are evaluated using the minimum inverted generational distance (IGD) on the aforementioned test function instances with 1000 decision variables [34]. A smaller IGD value indicates better overall performance of the algorithm in terms of convergence and diversity.

As shown in Table 1, Algorithm 1 utilizes random grouping as the grouping method. Algorithm 2 employs linear grouping as the grouping method. Algorithm 3 adopts the grouping method based on CRD and DD and all three algorithms utilize the improved MOPSO as the optimizer. Algorithm 4 combines the grouping method based on CRD and DD with the AADMOPSO optimizer. For each algorithm, 20 independent runs were conducted on each test function and the computational results were statistically analyzed to obtain the mean and variance. Table 1 presents the average and standard deviation of the IGD values for the four algorithms solving the four test problems.

The data in the table reveal that the proposed IALSCC algorithm, in most test functions, exhibit smaller mean and standard deviation of IGD values compared to the other algorithms. This indicates that IALSCC demonstrates superior convergence and diversity, as well as better algorithm stability. Thus, the performance of the algorithm has been validated.

We verify the high efficiency of the proposed algorithm IALSCC from two aspects. On the one hand, we illustrate based on the data that choosing a suitable threshold value can improve the efficiency of the algorithm. For each threshold, the algorithm was tested 20 times independently under the test function and the average value of the data was selected as the test value. Table 2 gives the IGD and running time of IALSCC for different thresholds. On the other hand, we demonstrate that the grouping method of the CC framework improves the efficiency of the algorithm. We test the performance and efficiency of the IALSCC algorithm using the grouping method and only using the improved MOPSO. Each algorithm was run independently 20 times on different test functions and the average value was selected as the test value. Table 3 gives the IGD values and running time of both algorithms.

From Table 2, it can be seen that as the threshold increases, the running time required by the algorithm becomes longer; especially after the threshold is specified as 200, the running time increases significantly, but at the same time, the performance of the algorithm is not significantly improved, so we choose the threshold of 100 to improve efficiency and at the same time can ensure good performance.

From Table 3, we can see that after using the grouping method in the CC frame-work, the performance of the two algorithms is not much different, but the efficiency of the IALSCC algorithms can be about doubled.

### 4.2. Numerical Validation

**Figure 6:**
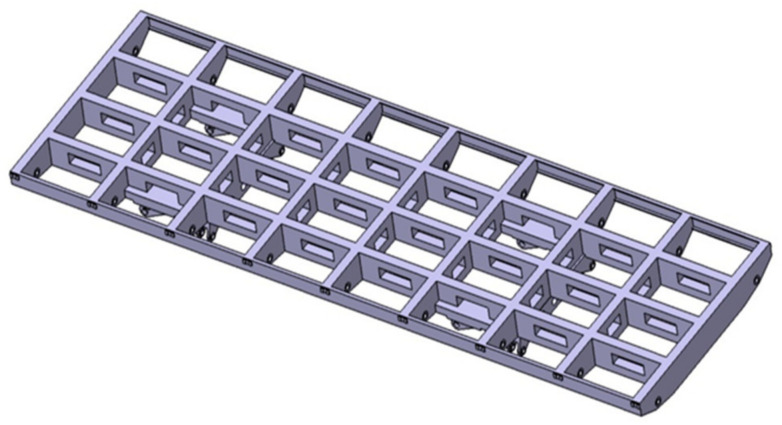
Antenna Truss Model.

To validate the generality of the IALSCC algorithm in the application of high-dimensional sensor placement, a simulation is conducted on a complex antenna truss structure. The finite element model of the antenna truss structure is established using the ABAQUS modeling and simulation software; it is modeled using 698,208 elements, including beam and solid. The Young’s modulus is E=7.03 GPa, the Poisson’s ratio is ν=0.3, and the density is $\rho =2700\;\mathrm{kg}/m^{3}$. The overall dimensions of the antenna truss structure are 5944 mm×1926 mm. The longitudinal beams consist of two side beams and three middle main beams. Both the main beams and side beams have a length of 653 mm. The main beams have a cross-sectional dimension of 260 mm×80 mm, while the side beams have a cross-sectional dimension of 120 mm×30 mm. The transverse beams have a length of 410mm and a cross-sectional dimension of 260 mm×80 mm. The hollow section in the main longitudinal beams has dimensions of 300 mm×100 mm and is located at the center of the main beams, as shown in Figure 6.

**Figure 7:**
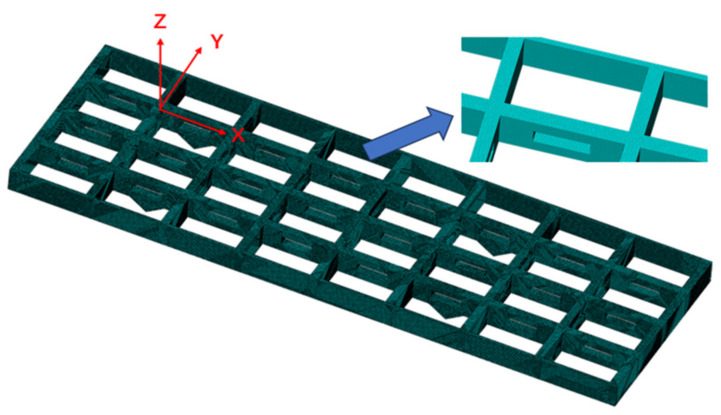
Truss Meshing and Coordinate System.

The coordinate system and grid division of the entire antenna truss structure are shown in Figure 7, with a grid spacing of 10 mm. Each longitudinal beam of the truss structure is divided into eight segments by ribs, resulting in a total of 40 inverse finite-element beam elements. Each inverse finite element is equipped with six strain sensors, leading to a total of forty by six sensors required for the entire truss structure. Therefore, one set of sensor layout scheme consists of two hundred forty sensors.

**Figure 8:**
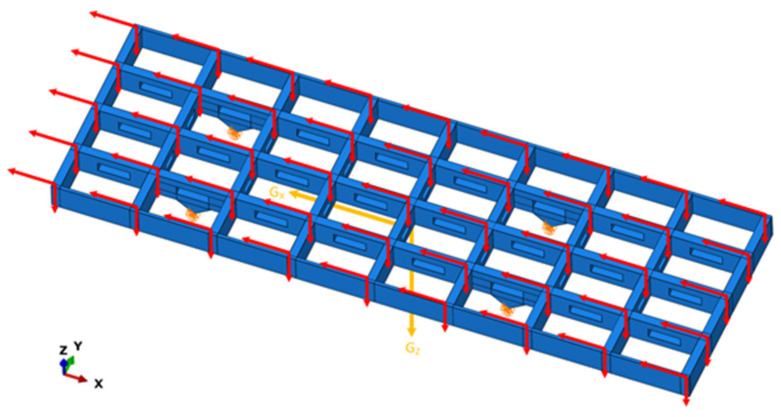
Forces and constraints of truss structures.

One side of the antenna truss is fixed to the bottom through a hinge, while the other side is connected to an adjustable top rod through a hinge, which is used to adjust the angle of the antenna array. Therefore, the constraints of the truss can be simplified to four hinge constraints that constrain the displacement degrees of freedom of the structure and release the rotation degrees of freedom of the structure. The truss structure is subjected to the gravity of the structure itself and concentrated forces at each node. The force and constraint conditions of the truss structure are shown in Figure 8. When the truss structure is tilted and the angle is changed, the distribution of gravity and concentrated forces in each direction will also change accordingly. Three typical operating conditions are selected for force analysis and the force conditions are shown in Table 4.

**Figure 9:**
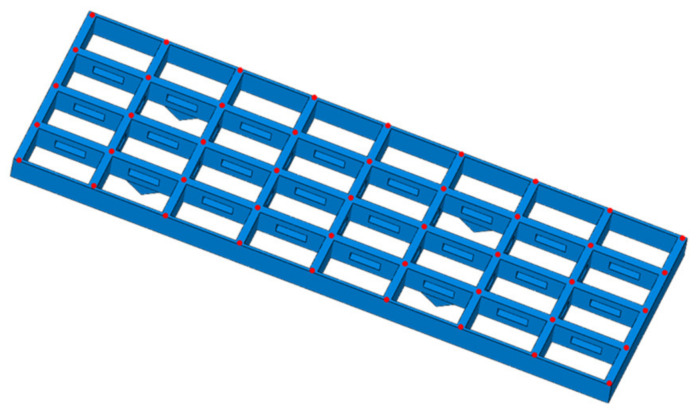
Check point distribution.

The strain information of nodes is extracted for three working conditions and the mapping relationship between sensor locations and the accuracy and robustness indicators is established based on the iFEM mentioned in Chapter 2. A high-dimensional bi-objective sensor layout optimization model is constructed. The validation points are uniformly selected throughout the truss structure, as shown in Figure 9. Additionally, since the primary deformation directions are X and Z, the accuracy indicator is calculated as the average RMSE in both directions.

The proposed IALSCC algorithm is applied for sensor layout optimization in the high-dimensional truss model, where the objective space consists of a series of nodes carrying strain information. The parameters are set as follows: the minimum number of groups ${\mathrm{sub}}_{\min}=10$, the maximum number of cycles of co-evolution is set to loop=10, the maximum number of subpopulation iterations is set to iter=60, and the number of divisions Di=40.

**Figure 10:**
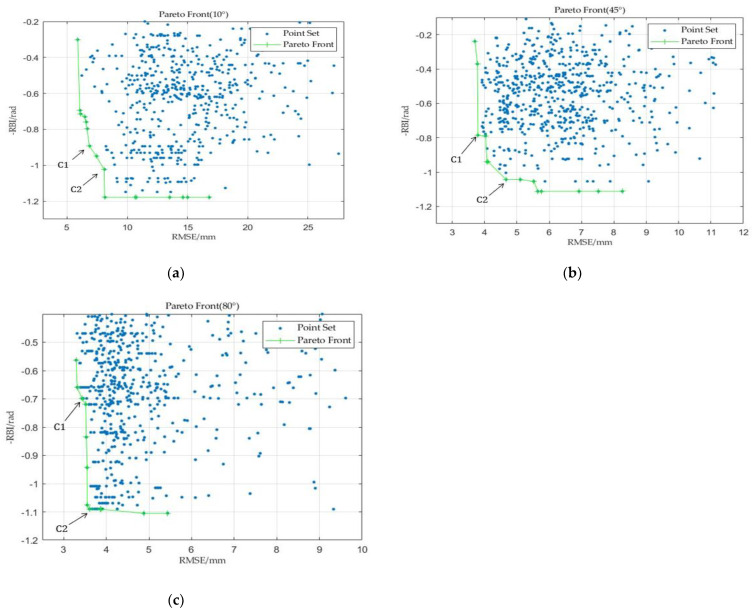
(**a**) PF of 10° working condition; (**b**) PF of 45° working condition; (**c**) PF of 80° working condition.

The optimization results are shown in Figure 10, where the blue particles represent the positions explored by the population and the green front represents the PF composed of non-dominated solutions. To visually display the PF, the figure only shows the solutions in the vicinity of the explored PF region. Each point in the figure contains the sensor position information under this scheme. From the PF shown in the figure, it can be inferred that the optimized sensor layout solution can achieve a high reconstruction accuracy.

Subsequently, typical solutions C1 and C2 are selected from the PF of the three operating conditions for the analysis of reconstruction accuracy and robustness. To visually demonstrate the reconstruction performance, the maximum displacement error ${\mathrm{ERR}}^{\max}$ is introduced, which is defined as follows:

(24) $$ERR^{max}=max\left|disp_{i}^{measurement}\left(X\right)-disp_{i}^{iFEM}\left(X\right)\right|,i=1,\cdots ,45,$$

where ${\mathrm{disp}}_{i}^{\mathrm{measurement}}$ represents the reference displacement of the i-th calibration point, extracted from the simulation software, and ${\mathrm{disp}}_{i}^{\mathrm{iFEM}}$ represents the displacement of the i-th calibration point reconstructed using the iFEM. Table 5 shows the maximum displacement error corresponding to the two sensor layout schemes and Max_disp is the maximum deformation.

Comparing the maximum displacement errors between the two schemes, under the condition of no interference when the antenna framework is tilted by 10° with the Z direction being the primary deformation direction and a maximum deformation of −55.6114 mm, the maximum displacement error for sensor layout scheme C1 is 7.0224 mm, which is better than the maximum displacement error of 8.3916 mm for sensor layout scheme C2. The maximum deformation in the X direction is 4.9752 mm, which can be considered negligible compared to the Z direction. When the antenna framework is tilted by 45° with maximum deformations of −7.2117 mm and −30.2659 mm in the X and Z directions, respectively, the maximum displacement errors for both directions under scheme C1 are 1.2756 mm and 4.3316 mm, which are superior to the maximum displacement errors of 1.8588 mm and 4.8450 mm under scheme C2. When the antenna framework is tilted by 80° with maximum deformations of −10.0236 mm and −11.4974 mm in the X and Z directions, respectively, the maximum displacement errors for both directions under scheme C1 are 3.0336 mm and 3.1346 mm, also outperforming the maximum displacement errors of 3.6807 mm and 3.6880 mm under scheme C2. The results demonstrate that the sensor layout scheme C1 exhibits higher reconstruction accuracy compared to scheme C2, consistent with the distribution of the reconstruction accuracy indicator *RMSE*, further reflecting the effectiveness of the reconstruction metrics.

In order to observe the stability of the two sensor layout schemes, 500 random disturbances are added to each sensor independently within the error range as specified in Equation (25); the class stiffness matrix will be recalculated after each addition of disturbances as it is only related to the sensor position, and then we perform iFEM reconstruction to evaluate the reconstruction error at this point. Finally, the data with the largest reconstruction error among the 500 experiments is selected and compared with Table 3 to verify the robustness metrics. The average maximum displacement error from 500 simulations is recorded and is presented in Table 4.

(25) $$∆x_{i}\in \left(-0.3,0.3\right),\;∆y_{i}\in \left(-0.05,0.05\right),\;∆z_{i}\in \left(-0.05,0.05\right),i=1,\cdots ,240;$$

According to Table 6, when the framework is lifted at 10° and disturbances are added, the maximum displacement errors in both directions for the C1 scheme increased from 1.9373 mm and 7.0224 mm to 2.7534 mm and 9.7508 mm, respectively, with a change of 0.8161 mm and 2.7284 mm. On the other hand, for C2 scheme the maximum displacement errors increase from 2.2805 mm and 8.3917 mm to 2.6112 mm and 9.0987 mm, respectively, with a change of 0.3307 mm and 0.707 mm. The data indicate that after introducing disturbances, the resistance ability of C2 scheme against disturbances is superior to that of C1 scheme and it also exhibits better reconstruction accuracy. Similar conclusions can be drawn for other working conditions.

When selecting a sensor scheme, it is necessary to consider the model’s requirements for both reconstruction accuracy and stability. Given that the antenna needs to operate under complex working conditions, it is more appropriate to choose sensor layout scheme C2, which exhibits better resistance against disturbances, while still meeting the accuracy requirements.

In conclusion, the algorithm proposed in this paper can be used to solve the problem of optimal sensor layout for complex structures, thus enabling health monitoring of large structures, such as antenna trusses, large aircraft wings, large bridges, and other engineering structures. When carrying out health monitoring of large structures, there is a greater demand for hardware demodulation capability and sensor failure detection capability, which is also a greater difficulty, and the pressure on the performance of the algorithm is also enormous if the inverse finite element is not reasonably divided in the face of a very complex structure. Our next research steps are as follows: (1). A more extensive study of grouping strategies in the CC framework; (2) Regarding the collaboration method within the CC framework, whether there is any space that can be further improved; (3) Whether there is a more reasonable method for threshold determination. An ongoing problem in the development of high-dimensional optimization algorithms is that it is difficult to simultaneously improve algorithmic performance and efficiency, and future research will also be devoted to solving this problem.

## 5. Conclusions

An optimization algorithm for sensor layout optimization problems in complex structures is proposed in this paper. Firstly, an inverse finite element model for multi-objective optimization of sensor layout is established based on reconstruction accuracy and robustness indicators. To address the issues of falling into local optima and low algorithm efficiency in previous algorithms when facing high-dimensional sensor optimization layout models, an IALSCC algorithm is proposed. It incorporates initialization strategies, adaptive region partitioning strategies, gbest selection, and particle update strategies to enhance algorithm diversity and introduces grouping methods based on the calculation of CRD and DD to improve algorithm efficiency. Then, the performance and efficiency of the IALSCC algorithm are verified in different comparative experiments. Finally, the proposed optimization algorithm IALSCC is applied to the deformation reconstruction of a complex antenna truss model, and the simulation results demonstrate its effectiveness in handling high-dimensional sensor layout optimization problems.

## Figures and Tables

**Table 1:** The mean and standard deviation of the IGD values of the four algorithms on the four test functions.

Test Function	Grouping Method	Random Grouping	Linear Grouping	CRD and DD	CRD and DD
	Optimizer	Improved MOPSO	Improved MOPSO	Improved MOPSO	AADMOPSO
ZDT1	Mean	2.668 × 10^−1^	8.803 × 10^−2^	**8.628 × 10^−2^**	2.32
	Std	2.376 × 10^−1^	8.394 × 10^−2^	**4.967 × 10^−2^**	6.377 × 10^−2^
ZDT2	Mean	1.282	6.916 × 10^−1^	**4.603 × 10^−1^**	3.726
	Std	8.706 × 10^−1^	4.881 × 10^−1^	**1.828 × 10^−1^**	1.839 × 10^−1^
ZDT3	Mean	1.887 × 10^−1^	6.280 × 10^−1^	**1.085 × 10^−1^**	3.096
	Std	7.045 × 10^−1^	1.106 × 10^−1^	**2.311 × 10^−2^**	1.189 × 10^−1^
ZDT6	Mean	8.818 × 10^−1^	8.793 × 10^−1^	**8.789 × 10^−1^**	8.798 × 10^−1^
	Std	1.723 × 10^−3^	1.487 × 10^−3^	1.579 × 10^−3^	**5.541 × 10^−4^**

**Table 2:** The effect of different thresholds on algorithm performance and efficiency (d = 1000).

IALSCC	Threshold	20	50	100	200
ZDT1	IGD	2.191 × 10^−1^	1.274 × 10^−1^	8.628 × 10^−2^	6.617 × 10^−2^
	Running time(s)	78.154	82.934	93.264	137.079
ZDT2	IGD	1.102	7.317 × 10^−1^	4.603 × 10^−1^	3.217 × 10^−1^
	Running time(s)	67.120	73.372	82.853	130.267
ZDT3	IGD	2.387 × 10^−1^	2.199 × 10^−1^	1.085 × 10^−1^	9.681 × 10^−2^
	Running time(s)	71.291	75.679	83.676	124.128
ZDT6	IGD	8.834 × 10^−1^	8.812 × 10^−1^	8.789 × 10^−1^	8.770 × 10^−1^
	Running time(s)	80.588	86.400	91.917	133.022

**Table 3:** Impact of grouping methods on algorithm performance and efficiency (d = 1000).

Optimization Algorithm	Test Function	ZDT1	ZDT2	ZDT3	ZDT6
IALSCC	IGD	8.628 × 10^−2^	4.603 × 10^−1^	1.085 × 10^−1^	8.789 × 10^−1^
	Running time(s)	93.264	82.853	83.676	91.917
Improved MOPSO	IGD	8.648 × 10^−2^	3.059 × 10^−1^	9.167 × 10^−2^	7.977 × 10^−1^
	Running time(s)	170.376	160.654	150.267	169.386

**Table 4:** The load applied by the antenna array (N).

Load	10°	45°	80°
Gravity component in X direction	−1700.898	−6926.89	−9649.91
Gravity component in Z direction	−9651.267	−6932.41	−1708.58
Concentration force in X direction	−4512.6	−18,377.5	−25,601.8
Concentration force in Z direction	−25,605.4	−18,392.1	−4532.98

**Table 5:** The maximum displacement error of a typical scheme (mm).

Condition	Direction	Max_disp	C1	C2
10°	X	4.9752	1.9373	2.2805
	Z	−55.6114	7.0224	8.3916
45°	X	−7.2117	1.2756	1.8588
	Z	−30.2659	4.3316	4.8450
80°	X	−10.0236	3.0336	3.6807
	Z	−11.4974	3.1346	3.6880

**Table 6:** The average maximum displacement error after adding 500 disturbances (mm).

Condition	Scheme	X	Z
10°	C1	2.7534	9.7508
	C2	2.6112	9.0987
45°	C1	2.3212	5.6491
	C2	2.4418	5.4987
80°	C1	4.1647	4.2683
	C2	4.3429	4.2425

## Data Availability

Not applicable.
